# *k*-Oligocarrageenan Promoting Growth of Hybrid Maize: Influence of Molecular Weight

**DOI:** 10.3390/molecules25173825

**Published:** 2020-08-22

**Authors:** Pham Trung San, Chau Minh Khanh, Huynh Hoang Nhu Khanh, Truong Anh Khoa, Nguyen Hoang, Le Thi Nhung, Nguyen Thi Kieu Trinh, Thanh-Danh Nguyen

**Affiliations:** 1 NhaTrang Institute of Technology Research and Application, Vietnam Academy of Science and Technology, NhaTrang City 57000, Vietnam; minhkhanhchau@gmail.com (C.M.K.); Khanhhuynh@nitra.vast.vn (H.H.N.K.); truonganhkhoa@nitra.vast.vn (T.A.K.); nguyenhoangnt77@nitra.vast.vn (N.H.); lethinhung@nitra.vast.vn (L.T.N.); Kieutrinh051194@gmail.com (N.T.K.T.); 2 Institute of Chemical Technology, Vietnam Academy of Science and Technology, HoChiMinh City 70000, Vietnam; danh5463bd@yahoo.com

**Keywords:** oligocarrageenan, promoter, molecule weight, hybrid maize (*Zea mays* L.), plant growth, grain yield

## Abstract

*k*-Oligocarrageenan (OC) is an effective biostimulator and a protector against disease infections for plants. However, the effect of OC molecular weight (MW) on plant growth is not fully understood. In this work, OCs with three different MWs (42, 17 and 4 kDa) was prepared by varying the degradation reaction time using ascorbic acid as a reagent. The product structure was confirmed by Fourier-transform infrared spectroscopy (FTIR) data. The growth promotion for maize (*Zea mays* L.) plants was investigated by foliar spray application of the prepared OCs. Field trials were carried out in two years, 2018 and 2019. The results showed that among treatments, OC with 4 kDa exhibited the best performance in both crop growth and grain yield parameters which indicated increases compared to the control in plant height (6.9–19.9%), length of ears (12.2%), diameter of ears (9.1%), fresh grain weight (17.8%), dry grain weight (20.0%) and grain yield (21.3%). Moreover, low MW OC augmented NP uptake in the plant growth while no effect on K uptake was observed. Therefore, OC with low MWs is potentially promising to apply as a promoter to enhance yield of crops.

## 1. Introduction

Plant growth is affected by various endogenous and exogenous factors such as temperature, light, enzymes and nutrient availability [1]. Biopromoters simulating plant growth are well known as biodegradable, biocompatible, biologically reactive and non-toxic molecules. They are particularly considered as chemical agents that enhance plant growth and crop yields [2]. Natural polysaccharides including alginate, chitosan and carrageenan are popular biopromoters possessing multidirectional effects in plant [3,4]. Furthermore, marine algae oligosaccharides could also simulate plant growth [5].

Carrageenan, the main cell-wall material in red algae or Rhodophyta, is a polysaccharide that contains repeating galactose units and 3,6-anhydrogalactose in both sulfated and non-sulfated types. There are three main classes, including κ-, ι- and λ-carrageenans that differ in their degree of sulfation [6]. Among them, κ-carrageenan extracted from *Kappaphycus alvarezii*, is frequently used for wide applications in food science, pharmaceutical technology and agriculture [7].

Oligocarrageenans (OCs) are commonly prepared by acidic hydrolysis of carrageenans using strong acids (HCl/H_2_SO_4_) [8], dilute HCl/LiCl [9,10] and gamma ray irrigation [11]. Their MWs could be controlled by varying the acid concentration, hydrolysis time and temperature. Although acidic hydrolysis uses simple, low cost and large scale techniques, strong acid reagents can damage the environment and produce toxic compounds. Therefore, green technologies are required for the fabrication of OCs with low MWs which could simulate plant growth through effectively enhancing photosynthesis, cell cycle and basal metabolism [12]. According to a literature review, some biopromoters have been applied for simulating maize growth. For instant, biopromoters derived from alfalfa could increase maize biomass, possibly as a result of improved nitrogen metabolism [13,14] or biopromoters made from various plant extracts could increase maize seed germination, seedling growth, radicle extension, and yield [15,16]. In this work, we have fabricated OCs with different MWs by acid hydrolysis using ascorbic acid as a reagent and applied them as biopromoters to simulate maize plant growth. Parameters such as plant growth, grain yield and NPK uptake have been investigated during two years, 2018 and 2019.

## 2. Results and Discussion

### 2.1. Synthesis and Characterizations of Oligocarrageenan

**Figure 1:**
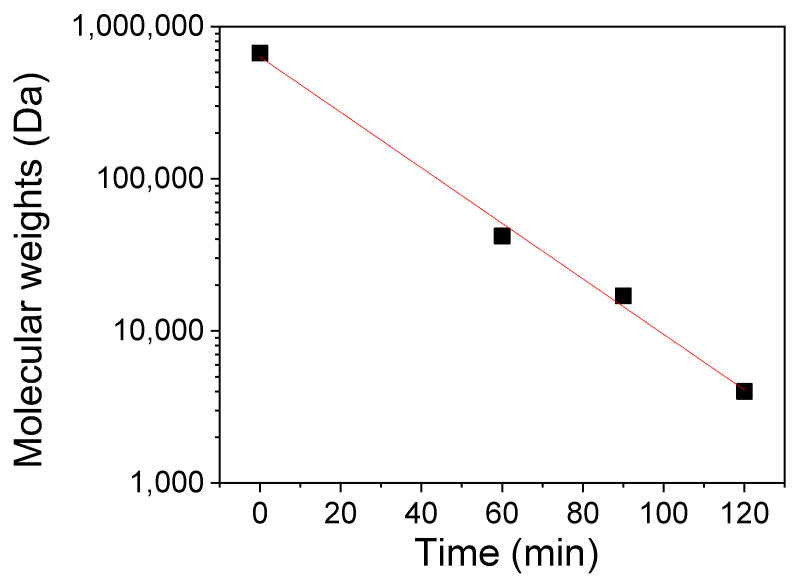
Influence of hydrolysis time on the molecular weight of oligocarrageenan.

The hydrolysis of κ-carrageenan in the presence of a natural acid (ascorbic acid) and H_2_O_2_ (2%) as a catalyst refluxed at 95 °C produces a degradation into OCs with different MWs. An obvious linear decrease of MW against reaction time is observed (Figure 1). The results agree well with previous reports [17,18]. The undegraded κ-carrageenan was determined to possess a MW of 670 kDa while the OCs obtained after the treatment with ascorbic acid for 60, 90 and 120 min had MW values of 42, 17 and 4 kDa, respectively. Sun et al. [19] have reported various methods for the degradation of carrageenan by using reactants including HCl or H_2_SO_4_ or/and H_2_O_2_. Our experiments used ascorbic acid as a reactant, which not only reduces significantly the risk to the environment but also plays a role as a nutrient component for plant growth.

**Figure 2:**
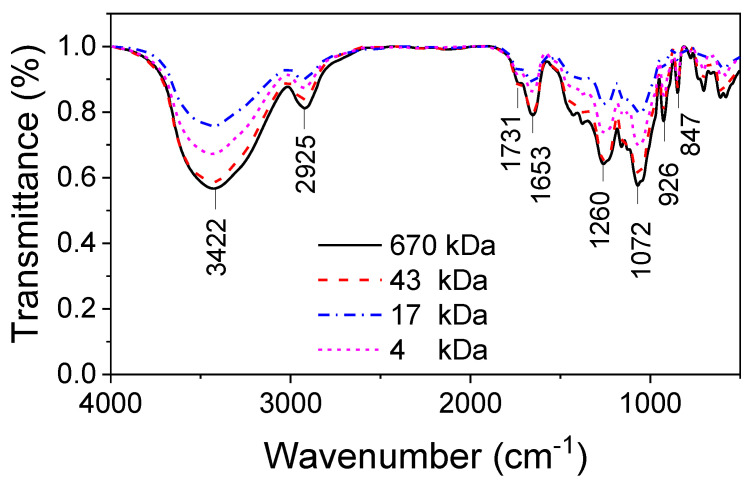
FTIR spectra of oligocarrageenans with different molecular weights.

FTIR analysis is a useful tool to elucidate the structural changes in the degraded OCs. The FTIR spectra of all OCs are shown in Figure 2. Similar absorption bands are observed in all spectra. The main absorption bands were observed at 3422, 2925, 1731, 1653, 1260, 1072, 926 and 847 cm^−1^. A broad band at 3422 cm^−1^ corresponds to the O-H stretching vibrations of glucoside molecules. The strong band at 2925 cm^−1^ is assigned to C-H stretching vibrations. Strong sharp bands at 1260 cm^−1^ and 847 cm^−1^ indicate the presence of the C-O-S=O groups of galactose-sulfate in the polysaccharide chain [20]. This result confirms no changes occurred in the structures of the OCs during hydrolysis, even for the OC with the lowest MW.

### 2.2. Growth Characters of Crop

Plant have evolved via various complex metabolic pathways to capture energy and form the metabolites required for their growth. Oligocarrageenan and carrageenans can promote the growth of crops by modulating different biochemical processes [21]. Many studies have shown that OCs are effective promoters for the growth of various crops. The different kinds of OCs such as κ, λ, ι-OCs have been shown to augment growth characteristics (e.g., plant height, leaf biomass, cell division and chlorophyll content) in tobacco by improving carbon fixation and nitrogen assimilation [12]. Saucedo et al. [22] found that treatment of *Pinus radiata* plants with OCs improved their growth parameters by inducing the accumulation of C, N and S. Gonzalez et al. [23] have reported that carrageenans induced increases of height and trunk diameter in *Eucalyptus globulus* plants. Among carrageenans, κ-carrageenan was found to be the most efficient crop promoter. However, the influence of oligocarrgenan MWs on the growth of maize plants as well as their nutrient uptake has not been investigated so far. In the present work, three samples of degraded OCs with different MWs (4, 17 and 42 kDa) were utilized to modulate the growth of hybrid maize during field experiments conducted over two years (2018 and 2019). The principle plant growth parameters (plant height, ear length, ear diameter) and crop yields (grain mass and grain yield) were explored. To study their effect on nutrient uptake ability, the contents of principle elements (N, P, K) in the main parts of the plants were evaluated by laboratory analysis.

**Figure 3:**
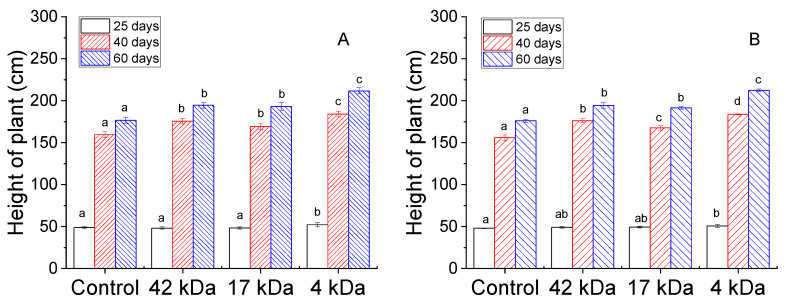
Influence of MWs on height of maize plant collected in 2018 (**A**) and 2019 (**B**). Bars represent mean values of three replicates ± standard deviation. Different letters indicate significant differences (*p* < 0.05).

The heights of maize plants were measured at 25, 40 and 60 days post-sowing in 2018 and 2019 as shown in Figure 3. The data of both years showed that the maize treated with OCs revealed an increase in plant height compared to the control plants and the OC with the lowest MW (4 kDa) induced the best crop growth. For instance, Figure 3A shows that the heights of control plants were 48.84, 159.56 and 176.58 cm at 25, 40 and 60 days post-sowing, respectively, while the heights of maize treated with 42 kDa OC were 175.70 cm and 194.58 cm, which corresponds to an increase of 10.1% and 10.2% at 40 and 60 days, respectively. On the other hand, the heights of plants treated with 4 kDa were 52.20, 184.06 and 211.69 cm at 25, 40 and 60 days, respectively, for an increase of 6.9%, 15.4% and 19.9% compared to the control plants and 8.2%, 4.8% and 8.8% compared to samples treated with 42 kDa MW OC. Results for 4 kDa OC were significantly different from each other, while crops treated with 42 kDa and 17 kDa OCs showed no significant differences in any of the parameters. Similar results were also obtained in 2019. This indicated that the OC small fraction might easily be absorbed by the plant and the existence of an absorption limitation for high MW OCs. However, the increase in the plant height 25 days after sowing was not significantly different between samples, in agreement with a previous experiment for oligochitosan. This can be due to the fact foliar application insignificantly affected intercellular CO_2_ concentrations in the photosynthesis of maize during the short time of growth [24].

Abad et al. [25] showed that application of fractionated radiation-modified OC solutions onto pechay plants induced plant growth in the decreasing order of 1 kDa > 3 kDa > 5 kDa. Application of other oligosaccharides such as oligochitosan have been known to enhance plant growth. Nge et al. [26] have reported a significant improvement in orchid growth when oligochitosans with low MWs were applied to orchid plant meristematic tissue. The results found that 1 kDa and 10 kDa oligochitosans were four times more effective compared to 100 kDa oligochitosan. Dzung et al. [27] applied oligochitosans with MWs in the range of 2.5–7.8 kDa to chili plants (*Capsicum frutescens*). The results showed that shoot weights increased in the order of applications 7.8 < 5.0 < 2.5 kDa.

**Figure 4:**
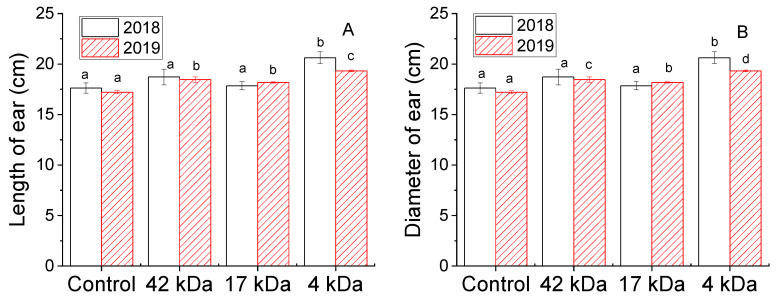
Influence of MWs on length of ear (**A**) and diameter of ear (**B**) collected in 2018 and 2019. Bars represent mean values of three replicates ± standard deviation. Different letters indicate significant differences (*p* < 0.05).

The effect of OC MWs on the growth parameters of maize ears is presented in Figure 4. It shows that the crops treated with OCs revealed an increase in length and diameter of ears and the data was slightly different between the two years. Experiments performed in 2018 showed that the mean length of ears increased in plants treated with OC 4 kDa by 17.1% compared to the control plant whereas this parameter was not different in plants treated with OCs 17 and 42 kDa compared to the control plant. Similar results also observed for the mean diameter of ears in the same year. Data collected in 2019 showed that the increases in mean length of ears were found to be 7.3%, 5.6% and 12.2% and the increases in mean diameter of ears were 5.5%, 3.2% and 9.1% when plants were treated with 42, 17 and 4 kDa OCs, respectively. Although different data are obtained between years which can be due to difference in mean rainfalls between 2018 and 2019, the highest increase in all parameters was clearly observed for crops treated with 4 kDa compared to the others. As a result, we can conclude that OC with a low MW induced an obvious enhancement in the growth of hybrid maize plants. The observed increase in growth of the plant treated with κ-carrageenan is in agreement with previous reports [12,28]. Umhaw et al. [29] applied binary mixtures of fertilizer (120-28-58 NPK) and κ-carrageenan to maize plant in The Philippines. Their data showed that the maximum increase in ear length is 9.0%, slightly lower than the result obtained from application of OC 4 kDa in the present work. Meanwhile, κ-carrageenan application also enhanced the growth characters of chickpea and maize plants [30].

### 2.3. Yield Parameters of Grain

**Figure 5:**
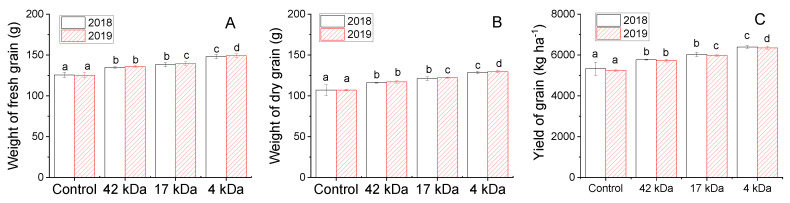
Influence of MWs on fresh (**A**) and dry (**B**) grain weights per ear and yield of grain (**C**) collected in 2018 and 2019. Bars represent mean values of three replicates ± standard deviation. Different letters indicate significant differences (*p* < 0.05).

Figure 5 describes effect of OC MWs on fresh grain weight, dry grain weight and grain yield obtained after harvests (about 100 days), collected in 2018 and 2019. The results show an increase in all grain yield variables after spraying the plant with OCs with different MWs. There is no significant difference between data collected in two years. For the results obtained in 2018, the mean fresh grain weight of control plants was 125.68 g ear^−1^ and that of plants treated with 42, 17 and 4 kDa was 134.66, 138.14 and 148.03 g ear^−1^ which corresponds to an increase of 7.1%, 12.5% and 17.8%, respectively. The mean dry grain weight of plants treated with OCs 42, 17 and 4 kDa was increased by 8.3%, 13.0% and 20.0%, respectively, compared to that of the control plants. A similarly increasing trend in grain weights with application of OCs was observed in 2019 and these increases were significantly different among treatments. Subsequently, grain yields of maize are significantly increased after treatment of crops with OCs. Mean values of grain yield increased by 8.27%, 13.01% and 20.04% in 2018 and 9.41%, 12.20% and 21.31% in 2019 when the crops treated with 42, 17 and 4 kDa OC, respectively. The highest increase in grain yields was also observed for application of the lowest OC of 4 kDa MW. This confirms that the absorption of OCs with low MWs can lead to better promotion of plant growth and grain yield after harvest. 

**Figure 6:**
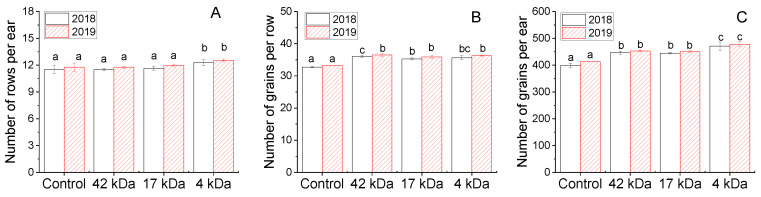
Influence of MWs on number of rows per ear (**A**) and number of grains per row (**B**) and number of grains per ear (**C**) collected in 2018 and 2019. Bars represent mean values of three replicates ± standard deviation. Different letters indicate significant differences (*p* < 0.05).

Ear characters including number of rows per ear, number of grains per row and number of grains per ear are presented in Figure 6. Similar results are observed for the two years. The number of rows per ear in plants treated with OC 42 and 17 kDa was not different from control plants whereas the treatment with OC 4 kDa evoked an increase of about 7.0% for both years. Mean values for the number of grains per row and number of grains per ear were considerably increased compared to the control plants and the maximum values were recorded in plants treated with OC 4 kDa. The increase in grain characters of plants treated with oligosaccharides possessing low MW can be related to more effective activation of enzymes such as nitrate reductase and glutamine synthetase which can yield high levels of protein in the treated plants [31,32]. Therefore, the determination of nutrient contents in the different parts of plant is particularly necessary to evaluate the enzyme activities and the metabolism in the plant.

### 2.4. Nutrient Uptake 

**Figure 7:**
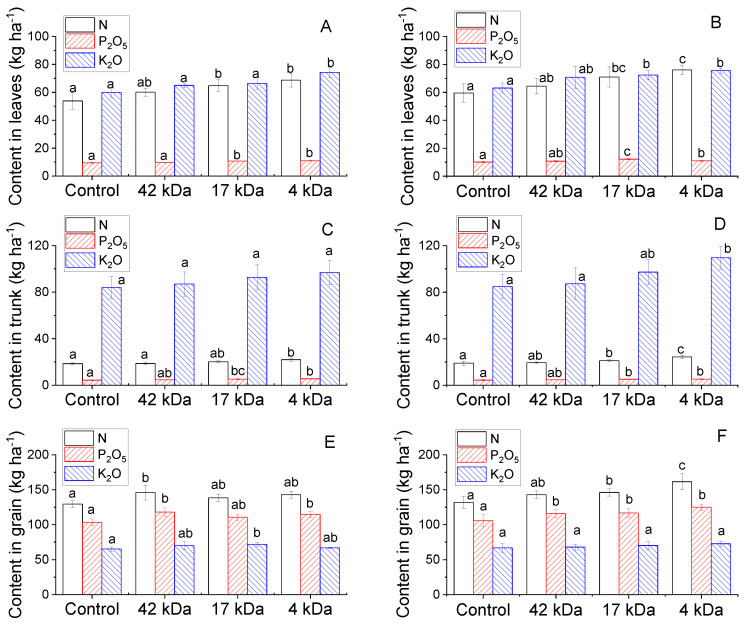
Influence of MWs on nutrient uptake determined in leaves (**A**,**B**), in trunk (**C**,**D**) and in grain (**E**,**F**) collected in 2018 (**left**) and 2019 (**right**). Bars represent mean values of three replicates ± standard deviation. Different letters indicate significant differences (*p* < 0.05).

Vigorous growth of maize under application of OCs can be related to an improved nutrient uptake ability in the plants. Figure 7 shows the content of NPK in various parts of maize analyzed in both years. An unambiguous increase of nitrogen and phosphorous contents in plants treated with OC 4 kDa was observed, while the effect of MW on potassium uptake was not clear in the growth of maize. For instant, the application of OC 4kDa in 2018 (Figure 7A,C,E) evoked nitrogen increases of 27.7%, 18.4% and 10.3% compared to the control plants as determined in leaves, trunk and grain, respectively, while the increase in phosphorus content was found to be 15.38%, 27.7% and 11.0%, respectively. However, the increase in potassium content in plant organs was not significant compared to the control plants, except for content in leaves of plants treated with 4 kDa OC. Similar results were observed in 2019. This was consistent with other reports that found the NP uptake closely related to tissue growth, yet K plays a major role in enzyme activation and maintaining cell osmotic potentials but is not incorporated into plant tissues [33].

Ning et al. [34] have also reported that K was necessary for vegetative growth pre-silking while NP content was important during the maturity period because grain development was mainly a process of carbohydrate deposition in maize. Therefore, it is clear that OCs having low MW can augment the NP uptake to grow plant tissues via a carbohydrate deposition process.

## 3. Materials and Methods 

### 3.1. Degradation of Carrageenan

Refined k-carrageenan extracted from the red *seaweed Kappaphycus alvarezii* was supplied by Sonhai Carrageenan Factory in Ninh Thuan province, Vietnam. OCs with various average MWs were prepared by hydrolysis of the k-carrageenan using ascorbic acid (0.4 M) and catalytic H_2_O_2_ (2%) at 90 °C for 60, 90 or 120 min.

### 3.2. Characterization of Oligocarrageenans

The average MWs were determined by the gel permeation chromatography (GPC) technique with an Agilent GPC-Addon Rev. B.01.01l. To study the possibly functional groups present in the OC chains, all samples were analyzed by Fourier transform infrared (FTIR) spectroscopy. The FTIR spectra were recorded on a Tensor 27 instrument (Bruker, Karlsruhe, Germany) over a wavelength number range of 500–4000 cm^−1^.

### 3.3. Field Procedures

For the field trials, the OCs with different MWs (4-42 kDa) were used to evaluate growth and physiological activity of hybrid maize (*Zea mays* L.). The experiments were carried out in CuMgar, Daklak province, Vietnam (12°47′31′N and 108°04′35′E) at 500 m of height over the seawater level for two years (2018 and 2019). Basal soil was used for field trial and characters of experimental soil including nitrogen (0.15 ± 0.02%), phosphorous (0.192 ± 0.032%), potassium (0.098 ± 0.023), pH (5.08 ± 0.203), humidity (33.58 ± 4.029%), humus (3.33 ± 0.769%) and bacteria (3.56 × 10^5^ ± 1.56 × 10^5^ CFU mg^−1^) were analysized. Meteorological data for each month including air temperature and total rainfall in two years 2018 and 2019 collected by the Tay Nguyen regional hydrometeorological center and is listed in Table S1 (Supplementary Materials).

**Figure 8:**
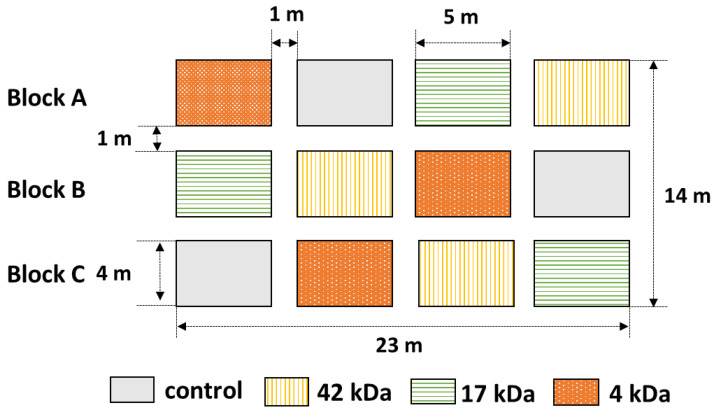
Field experiment design for biostimulator treatment of hybrid maize.

The field experiments were conducted under a randomized complete block design with four treatments and three repetitions as shown in Figure 8. An area of land (14 m × 23 m) was selected and divided into three equal blocks named Block A, Block B and Block C. Each block was further divided into four plots with a distance between two plots of one meter so that each plot covered 20 m^2^ (4 m × 5 m). Four biostimulator treatments were randomly applied to the four plots in each block. Each plot containing 68 plants was divided into four rows so that there is 17 plants in each row. The space between plants is about 0.25 m. The different promoters were applied as four treatments including: (1) only water (control), (2) OC with MW of 42 kDa (42 kDa), (3) OC with MW of 17 kDa (17 kDa) and (4) OC with MW of 4 kDa (4 kDa). The blocks were defined as three replicates.

The sowing process was carried out by the dibbling method placing two sown seeds per hill later thinned to one plant per hill. NPK fertilizer was applied in the form of urea (46.3% nitrogen), calcium dihydrophosphate (15–17% phosphorous) and potassium chloride (50–60% potassium), respectively. The different fertilizer contents were irrigated at various periods during the plant growth process. OCs with different MWs (at the same concentration) were applied by foliar spray. Cattle manure (10 ton ha^−1^), nitrogen (40 kg ha^−1^) and phosphorous (80 kg ha^−1^) fertilizers were applied at 20 days pre-sowing. Spray of OCs (100 ppm), and irrigation of nitrogen (40 kg ha^−1^) and potassium (40 kg ha^−1^) fertizers were firstly performed when the maize had 4–5 leaves (~15 days post-sowing). Secondly, a spray of OCs (100 ppm), and irrigation of nitrogen (80 kg ha^-1^) and potassium (40 kg ha^−1^) fertilizer were applied when the maize had 7–9 leaves (~30 days post-sowing). Finally, a spray of OCs (100 ppm), and irrigation of nitrogen (80 kg ha^−1^) and potassium (40 kg ha^−1^) fertilizers were applied at 10–15 days pre-anthesis (~50 days post-sowing). Therefore, a total amount of nitrogen of 240 kg ha^−1^, phophorus 80 kg ha^−1^ and potassium 120 kg ha^−1^ was applied to ensure the physiological stages of the crops. The maize was kept free of weeds to avoid weed-crop competition. The crop was manually harvested on December 2018 and December 2019.

The crop traits and grain yields were evaluated via the national standard guidance (QCVN 01-56:2011/BNNPTNT) as follows: (1) plant height of 30 random crops, which planted in two central rows of the plots, were measured as the distance from ground level to the lowest branch of corn tassel at 25, 40 and 60 days post-sowing; (2) length and diameter of the ears were averagely determined from 30 ears (the lowest ear for each plant was used); (3) number of grains per ear was determined by counting the total number of grains in 30 ears and then dividing by the number of the ears; (4) number of grain rows per ear in 30 ears was identified as the average number of grain rows in an ear; (5) number of grains per grain row in 30 ears was calculated by the number of grains for only a mean row; (6) grain yield (kg ha^−1^) were calculated by Equation (1):

(1) $$Yield=\frac{m_{1}m_{2}}{S_{0}m_{0}}\times 10^{3}$$

where *m*_1_ is fresh corn mass of two central rows in each plot; *m*_2_ is dry grain mass of 30 plants (humidity < 14%); *m*_0_ is fresh corn mass of 30 plants; *S*_0_ is land area of two central rows (7 m^2^).

Analysis of variance and mean comparisons of the collected data were performed Duncan’s Multiple Range Test (DMRT) software package. 

### 3.4. Laboratory Analysis

The NPK uptakes in different parts of the crop including leaves, trunk and grains were estimated by multiplying of dry biomass weight. Fifteen random plants (100 days old) in each plot were selected for analysis. The samples were dried in air at 75 °C for 72 h and then grounded into a fine powder. The powder was digested in a solution of H_2_SO_4_ (98%) containing H_2_O_2_ (30%). The nitrogen content was estimated by the Kjeldahl method while phosphorus and potassium content were analysed by a spectrophotometric method, and atomic absorption spectrometry (AAS), respectively.

## 4. Conclusions

Foliar spraying of OCs on maize plants promotes growth and enhances grain yield. The results supported the notions that application of OC with low MW (4 kDa) induced significant increases in plant growth and the grain yield parameters of maize plants. Furthermore, the present work showed that OC with low MW can augment NP uptake but not K uptake during maize plant growth. This result provides particularly important information for famers in development of green agriculture and also promotes investigation of the effect of application of OCs on other crops in the future.

## Supplementary Materials

The Supplementary Materials are available online. Table S1: Meteorological data for each month in Daklak province, Vietnam collected in two years 2018 and 2019 collected, Table S2: Influence of molecular weight of oligocarrageenan on maize plant height and maize ear in two years, Table S3: Influence of molecular weight of oligocarrageenan on parameters of ear growth and crop yield, Table S4: Influence of molecular weight of oligocarrageenan on NPK uptakes (kg ha^−1^) by maize plant in two years.

## References

[1] Lau O.S., Deng X.W. (2010). Plant hormone signaling lightens up: Integrators of light and hormones. Curr. Opin. Plant Boil..

[2] Liu W., Stewart C.N. (2016). Plant synthetic promoters and transcription factors. Curr. Opin. Biotechnol..

[3] Liu J., Kennedy J.F., Zhang X., Heng Y., Chen W., Chen Z., Wu X., Wu X. (2020). Preparation of alginate oligosaccharide and its effects on decay control and quality maintenance of harvested kiwifruit. Carbohydr. Polym..

[4] Digruber T., Sass L., Cseri A., Paul K., Nagy A.V., Remenyik J., Molnar I., Vass I., Toldi O., Gyuricza C. (2018). Stimulation of energy willow biomass with triacontanol and seaweed extract. Ind. Crop. Prod..

[5] Laporte D., Vera J., Chandía N.P., Zúñiga E.A., Matsuhiro B., Moenne A. (2006). Structurally unrelated algal oligosaccharides differentially stimulate growth and defense against tobacco mosaic virus in tobacco plants. Environ. Boil. Fishes.

[6] Zia K.M., Tabasum S., Nasif M., Sultan N., Aslam N., Noreen A., Zuber M. (2017). A review on synthesis, properties and applications of natural polymer based carrageenan blends and composites. Int. J. Boil. Macromol..

[7] Usman A., Khalid S., Usman A., Hussain Z., Wang Y., Zia K.M., Zuber M., Ali M. (2017). Algal Polysaccharides, Novel Application, and Outlook. Algae Based Polymers, Blends, and Composites.

[8] Kamińska-Dwórznicka A., Antczak A., Samborska K., Lenart A. (2015). Acid hydrolysis of kappa-carrageenan as a way of gaining new substances for freezing process modification and protection from excessive recrystallisation of ice. Int. J. Food Sci. Technol..

[9] Hjerde T., Smidsrød O., Stokke B.T., Christensen B.E. (1998). Acid Hydrolysis of κ- and ι-Carrageenan in the Disordered and Ordered Conformations: Characterization of Partially Hydrolyzed Samples and Single-Stranded Oligomers Released from the Ordered Structures. Macromolecules.

[10] Singh S.K., Jacobsson S.P. (1994). Kinetics of acid hydrolysis of κ-carrageenan as determined by molecular weight (SEC-MALLSRI), gel breaking strength, and viscosity measurements. Carbohydr. Polym..

[11] Abad L.V., Relleve L.S., Racadio C.D.T., Aranilla C.T., De La Rosa A.M. (2013). Antioxidant activity potential of gamma irradiated carrageenan. Appl. Radiat. Isot..

[12] Castro J., Vera J., Gonzalez A., Moenne A. (2011). Oligo-Carrageenans Stimulate Growth by Enhancing Photosynthesis, Basal Metabolism, and Cell Cycle in Tobacco Plants (var. Burley). J. Plant Growth Regul..

[13] Ertani A., Schiavon M., Muscolo A., Nardi S. (2012). Alfalfa plant-derived biostimulant stimulate short-term growth of salt stressed Zea mays L. plants. Plant Soil.

[14] Ertani A., Cavani L., Pizzeghello D., Brandellero E., Altissimo A., Ciavatta C., Nardi S. (2009). Biostimulant activity of two protein hydrolyzates in the growth and nitrogen metabolism of maize seedlings. J. Plant Nutr. Soil Sci..

[15] Ziosi V., Zandoli R., Vitali F., Nardo A.D. (2013). Folicist, a biostimulant based on acetyl- thioproline, folic acid and plant extracts, improves seed germination and radile extension. Acta Hortic..

[16] Layek J., Das A., Ramkrushna G.I., Trivedi K., Yesuraj D., Chandramohan M., Kubavat D., Agarwal P.K., Ghosh A. (2015). Seaweed sap: A sustainable way to improve productivity of maize in North-East India. Int. J. Environ. Stud..

[17] Sun T., Tao H., Xie J., Zhang S., Xu X. (2010). Degradation and antioxidant activity of κ-carrageenans. J. Appl. Polym. Sci..

[18] Hjerde T., Stokke B.T., Smidsrød O., Christensen B.E. (1998). Free-radical degradation of triple-stranded scleroglucan by hydrogen peroxide and ferrous ions. Carbohydr. Polym..

[19] Sun Y., Yang B., Wu Y., Liu Y., Gu X., Zhang H., Wang C., Cao H., Huang L., Wang Z. (2015). Structural characterization and antioxidant activities of κ-carrageenan oligosaccharides degraded by different methods. Food Chem..

[20] Pereira L., Van De Velde F. (2011). Portuguese carrageenophytes: Carrageenan composition and geographic distribution of eight species (Gigartinales, Rhodophyta). Carbohydr. Polym..

[21] Shukla P.S., Borza T., Critchley A.T., Prithiviraj B. (2016). Carrageenans from Red Seaweeds As Promoters of Growth and Elicitors of Defense Response in Plants. Front. Mar. Sci..

[22] Saucedo S., Contreras R.A., Moenne A. (2015). Oligo-carrageenan kappa increases C, N and S assimilation, auxin and gibberellin contents, and growth in Pinus radiata trees. J. For. Res..

[23] González A., Contreras R.A., Moenne A. (2013). Oligo-Carrageenans Enhance Growth and Contents of Cellulose, Essential Oils and Polyphenolic Compounds in Eucalyptus globulus Trees. Molecules.

[24] Khan W., Prithiviraj B., Smith D. (2002). Effect of Foliar Application of Chitin and Chitosan Oligosaccharides on Photosynthesis of Maize and Soybean. Photosynthetica.

[25] Abad L.V., Aurigue F.B., Relleve L.S., Montefalcon D.R.V., Lopez G.E.P. (2016). Characterization of low molecular weight fragments from gamma irradiated κ-carrageenan used as plant growth promoter. Radiat. Phys. Chem..

[26] Nge K.L., Nwe N., Chandrkrachang S., Stevens W.F. (2006). Chitosan as a growth stimulator in orchid tissue culture. Plant Sci..

[27] Dzung P.D., Van Phu D., Du B.D., Ngoc L.S., Duy N.N., Hiet H.D., Nghia D.H., Thang N.T., Van Le B., Hien N.Q. (2017). Effect of foliar application of oligochitosan with different molecular weight on growth promotion and fruit yield enhancement of chili plant. Plant Prod. Sci..

[28] González A., Gutierrez-Cutiño M., Moenne A. (2014). Oligo-Carrageenan Kappa-Induced Reducing Redox Status and Increase in TRR/TRX Activities Promote Activation and Reprogramming of Terpenoid Metabolism in Eucalyptus Trees. Molecules.

[29] Umhaw G.P., Naval R.C., Dolojan F.M., Abella M.E.S., Hizon M.G.S., Mabborang S.A. (2020). Effects of radiation-modified kappa-carrageenan supplementation in corn (*Zea mays* L.). J. Crit. Rev..

[30] Bi F., Iqbal S., Arman M., Ali A., Mahmood Q. (2011). Carrageenan as an elicitor of induced secondary metabolites and its effects on various growth characters of chickpea and maize plants. J. Saudi Chem. Soc..

[31] Zhang Y., Zhang G., Liu L., Zhao K., Wu L., Hu C., Di H. (2011). The role of calcium in regulating alginate-derived oligosaccharides in nitrogen metabolism of Brassica campestris L. var. utilis Tsen et Lee. Plant Growth Regul..

[32] Rengasamy K.R.R., Kulkarni M.G., Stirk W.A., Van Staden J. (2015). Eckol Improves Growth, Enzyme Activities, and Secondary Metabolite Content in Maize (Zea mays cv. Border King). J. Plant Growth Regul..

[33] Carroll M.J., Slaughter L.H., Krouse J.M. (1907). Turgor Potential and Osmotic Constituents of Kentucky Bluegrass Leaves Supplied with Four Levels of Potassium. Agron. J..

[34] Ning P., Liao C., Li S., Yu P., Zhang Y., Li X., Li C. (2012). Maize cob plus husks mimics the grain sink to stimulate nutrient uptake by roots. Field Crop. Res..

