# Supplementary material for: k-Oligocarrageenan Promoting Growth of Hybrid Maize: Influence of Molecular Weight
Source: Molecules. 2020 Aug 22;25(17):3825. doi: 10.3390/molecules25173825 (PMC7503339; doi:10.3390/molecules25173825)
Supplement: Supplementary file 1 [file molecules-25-03825-s001.pdf]

k-Oligocarrageenan promoting growth of hybrid maize: Influence of molecular weight.

Pham Trung San[1]*, Chau Minh Khanh[1], Huynh Hoang Nhu Khanh[1], Truong Anh Khoa[1], Nguyen Hoang[1], Le Thi Nhung[1], Nguyen Thi Kieu Trinh[1], Thanh-Danh Nguyen[2]

[1]NhaTrang Institute of Technology Research and Application, Vietnam Academy of Science and Technology, NhaTrang City, Vietnam.

[2]Institute of Chemical Technology, Vietnam Academy of Science and Technology, HoChiMinh City, Vietnam.

***Corresponding author:** Pham Trung San. Email: phamtrungsan@nitra.vast.vn

Supplementary Data

Table S1. Meteorological data for each month in Daklak province, Vietnam collected in two years 2018 and 2019 collected by the Tay Nguyen regional hydrometeorological center.

Month	2018		2019	
	Rainfall (mm)	Temp. (ºC)	Rainfall (mm)	Temp. (ºC)
1	-	22.0	25.4	21.9
2	-	22.0	1.6	21.8
3	33.1	24.7	7.0	24.1
4	105.1	26.1	106.3	25.8
5	204.1	26.3	346.7	25.7
6	228.4	25.9	208.4	25.2
7	238.0	24.9	222.0	24.8
8	298.7	25.5	165.0	24.5
9	391.0	25.3	194.8	24.7
10	22.3	24.4	198.1	24.6
11	87.2	24.0	100.0	23.8
12	22.7	21.4	47.0	23.4

Table S2. Influence of molecular weight of oligocarrageenan on maize plant height and maize ear in two years

Treatments	Plant height (cm)			Maize ear size (cm)	
	**25 days**	**40 days**	**60 days**	**Length**	**Diameter**
			**2018**		
Control (water)	48.84[a]	159.56[a]	176.58[a]	17.62[a]	4.16[a]
42 kDa	48.23[a]	175.70[b]	194.58[b]	18.72[a]	4.46[a]
17 kDa	48.32[a]	169.20[b]	193.01[b]	17.85[a]	4.41[a]
4 kDa	52.20[b]	184.06[c]	211.69[c]	20.62[b]	5.02[b]
CV (%)	*3.42*	*2.02*	*1.98*	*3.14*	*4.09*
LSD, 0.05	*3.04*	*6.24*	*6.90*	*1.05*	*0.33*
			**2019**		
Control (water)	48.07[a]	155.90[a]	176.03[a]	17.21[a]	4.38[a]
42 kDa	49.02[ab]	176.22[b]	194.40[b]	18.47[b]	4.62[c]
17 kDa	49.31[ab]	167.68[c]	191.52[b]	18.18[b]	4.52[b]

4 kDa	50.83^{b}	183.74^{d}	212.33^{c}	19.32^{c}	4.78^{d}
CV (%)	*2.19*	*1.38*	*1.10*	*0.88*	*1.00*
LSD, $_{0.05}$	*1.94*	*4.23*	*3.83*	*0.29*	*0.08*

Different letters indicate significant differences (P < 0.05).

Table S3. Influence of molecular weight of oligocarrageenan on parameters of ear growth and crop yield.

**Treatments**	**Number of rows per ear**	**Number of grains per row**	**Number of grains per ear**	**Fresh grains weight (g)**	**Dry grains weight (g)**	**Grain yield (kg ha^{-1})**
			**2018**			
Control (water)	11.51^{a}	32.73^{a}	398.30^{a}	125.68a	107.16^{a}	5329
42 kDa	11.50^{a}	36.03^{c}	446.83^{b}	134.66^{b}	116.02^{b}	5770
17 kDa	11.64^{a}	35.26^{b}	444.54^{b}	138.14^{b}	121.10^{b}	6022
4 kDa	12.31^{b}	35.63^{bc}	471.32^{c}	148.03^{c}	128.63^{c}	6397
CV (%)	*2.56*	*1.03*	*2.16*	*1.82*	*2.92*	
$LSD_{0.05}$	*0.54*	*0.65*	*17.06*	*4.48*	*6.19*	
			**2019**			
Control (water)	11.78^{a}	33.26^{a}	413.06^{a}	125.31^{a}	106.97^{a}	5235
42 kDa	11.78^{a}	36.48^{b}	453.15^{b}	135.85^{b}	117.04^{b}	5727
17 kDa	11.97^{a}	35.91^{b}	450.77^{b}	139.36^{b}	122.17^{c}	5978

4 kDa	12.54^{b}	36.29^{b}	478.04^{c}	149.34^{c}	129.77^{d}	6350
CV (%)	*2.08*	*1.00*	*1.12*	*1.72*	*1.06*	
$LSD_{0.05}$	*0.45*	*0.63*	*9.03*	*4.24*	*2.27*	

Different letters indicate significant differences ($P < 0.05$).

Table S4. Influence of molecular weight of oligocarrageenan on NPK uptakes (kg ha^{-1}) by maize plant in two years

Treatments	Leaves			Trunk			Grains		
	N	P_2O_5	K_2O	N	P_2O_5	K_2O	N	P_2O_5	K_2O
**2018**									
Control (water)	53.81^{a}	9.49^{a}	60.01^{a}	18.53^{a}	4.33^{a}	83.97^{a}	129.50^{a}	103.34^{a}	65.03^{a}
42 kDa	60.12^{ab}	9.84^{a}	64.98^{a}	18.73^{a}	4.65^{ab}	86.93^{a}	145.88^{b}	118.03^{b}	70.03^{ab}
17 kDa	64.75^{b}	10.75^{b}	66.29^{a}	20.15^{ab}	5.18^{bc}	92.68^{a}	138.32^{ab}	110.61^{ab}	71.87^{b}
4 kDa	68.70^{b}	10.95^{b}	74.30^{b}	21.95^{b}	5.53^{b}	96.77^{a}	142.92^{ab}	114.69^{b}	66.68^{ab}
CV (%)	*7.42*	*2.77*	*5.04*	*5.19*	*6.74*	*11.39*	*4.95*	*4.49*	*4.86*
LSD, $_{0.05}$	*8.24*	*0.51*	*6.01*	*1.85*	*0.60*	*18.42*	*12.38*	*9.01*	*5.97*
**2019**									
Control (water)	59.55^{a}	10.07^{a}	63.10^{a}	19.01^{a}	4.45^{a}	84.82^{a}	131.74^{a}	105.58^{a}	66.71^{a}
42 kDa	64.46^{ab}	10.72^{ab}	70.80^{ab}	19.43^{ab}	4.69^{ab}	87.23^{a}	142.79^{ab}	115.76^{b}	67.81^{a}
17 kDa	71.01^{bc}	12.19^{c}	72.41^{b}	21.19^{b}	5.22^{b}	97.30^{ab}	146.10^{b}	116.84^{b}	70.10^{a}
4 kDa	76.10^{c}	10.94^{b}	75.61^{b}	24.36^{c}	5.25^{b}	109.56^{b}	161.43^{c}	124.92^{b}	72.59^{a}

CV (%)	*8.60*	*3.51*	*6.69*	*5.67*	*8.03*	*11.90*	*5.65*	*5.61*	*6.86*
LSD, $_{0.05}$	*10.47*	*0.69*	*8.47*	*2.14*	*0.71*	*20.25*	*14.78*	*11.66*	*8.53*

Different letters indicate significant differences ($P < 0.05$).
